# Prediction of severe community-acquired pneumonia: a systematic review and meta-analysis

**DOI:** 10.1186/cc11447

**Published:** 2012-07-27

**Authors:** Christophe Marti, Nicolas Garin, Olivier Grosgurin, Antoine Poncet, Christophe Combescure, Sebastian Carballo, Arnaud Perrier

**Affiliations:** 1 Department of Internal Medicine, Rehabilitation and Geriatrics, University Hospitals of Geneva and Geneva Faculty of Medicine, Rue Gabrielle Perret-Gentil 4, 1211 Geneva 14, Switzerland; 2 Division of Internal Medicine, Regional Hospital of Chablais, Route de Morgins, Monthey, 1870 Switzerland; 3 Department of Health and Community Medicine, University Hospitals of Geneva and Geneva Faculty of Medicine, Rue Gabrielle Perret-Gentil 4, 1211 Geneva 14, Switzerland

## Abstract

**Introduction:**

Severity assessment and site-of-care decisions for patients with community-acquired pneumonia (CAP) are pivotal for patients' safety and adequate allocation of resources. Late admission to the intensive care unit (ICU) has been associated with increased mortality in CAP. We aimed to review and meta-analyze systematically the performance of clinical prediction rules to identify CAP patients requiring ICU admission or intensive treatment.

**Methods:**

We systematically searched Medline, Embase, and the Cochrane Controlled Trials registry for clinical trials evaluating the performance of prognostic rules to predict the need for ICU admission, intensive treatment, or the occurrence of early mortality in patients with CAP.

**Results:**

Sufficient data were available to perform a meta-analysis on eight scores: PSI, CURB-65, CRB-65, CURB, ATS 2001, ATS/IDSA 2007, SCAP score, and SMART-COP. The estimated AUC of PSI and CURB-65 scores to predict ICU admission was 0.69. Among scores proposed for prediction of ICU admission, ATS-2001 and ATS/IDSA 2007 scores had better operative characteristics, with a sensitivity of 70% (CI, 61 to 77) and 84% (48 to 97) and a specificity of 90% (CI, 82 to 95) and 78% (46 to 93), but their clinical utility is limited by the use of major criteria.

ATS/IDSA 2007 minor criteria have good specificity (91% CI, 84 to 95) and moderate sensitivity (57% CI, 46 to 68). SMART-COP and SCAP score have good sensitivity (79% CI, 69 to 97, and 94% CI, 88 to 97) and moderate specificity (64% CI, 30 to 66, and 46% CI, 27 to 66). Major differences in populations, prognostic factor measurement, and outcome definition limit comparison. Our analysis also highlights a high degree of heterogeneity among the studies.

**Conclusions:**

New severity scores for predicting the need for ICU or intensive treatment in patients with CAP, such as ATS/IDSA 2007 minor criteria, SCAP score, and SMART-COP, have better discriminative performances compared with PSI and CURB-65. High negative predictive value is the most consistent finding among the different prediction rules. These rules should be considered an aid to clinical judgment to guide ICU admission in CAP patients.

## Introduction

Community-acquired pneumonia (CAP) is a major health problem. In the United States, 500,000 adults are hospitalized annually for CAP [1], of whom 10% to 20% are admitted to the intensive care unit (ICU) [2]. Because site of care is a major determinant of costs, clinical prediction rules have been developed to identify patients with low mortality who can be safely treated as outpatients [3].

Since 1993, efforts have been made to identify severe community-acquired pneumonia (SCAP) requiring admission to the ICU because the ICU is an expensive and scarce resource. Concurrently, delay in ICU admission of CAP patients has been shown to be associated with increased mortality [4-6]. In 1993, the American Thoracic Society (ATS) proposed a definition of severe CAP requiring ICU admission [7]. Since then, these criteria have been updated twice [8,9], and various clinical prediction rules have been developed to predict SCAP [10-12]. In addition to the variety of the prediction rules, the assessment of their validity is further hampered by the absence of a unique definition of SCAP and the inclusion of ICU admission or intensive treatment in the definition, which exposes prediction rules for SCAP to incorporation bias [13].

The goal of our search was to review systematically the performance of existing clinical prediction rules to identify in the Emergency Department patients with CAP requiring ICU admission or intensive treatment.

## Materials and methods

### Search strategy and study selection

We systematically searched Medline, Embase, and the Cochrane Controlled Trials registry by using the following key words: *community-acquired pneumonia *AND (*decision tree *OR *clinical prediction rule *OR *clinical prediction score *OR *clinical decision rule *OR *clinical decision score *OR *management studies *OR *outcome studies *OR *ICU admission *OR *ICU need *OR *invasive management *OR *severity assessment*). The search was performed for articles in English, French, Italian, Spanish, and German languages and limited to articles with an abstract and completed on the first of March 2012. To ensure a comprehensive literature search, we examined reference lists from retrieved articles and reference literature (guidelines and systematic reviews) and questioned experts in CAP for possible missing studies.

### Study inclusion and data extraction

Eligible studies were prospective or retrospective studies evaluating clinical prediction rules in adult immunocompetent patients with CAP to predict the need for ICU admission, intensive treatment, or early mortality (< 14 days). The evaluation had to be performed during the first 24 hours after hospital admission. Studies addressing specific patient subgroups based on etiology or age were excluded. A prediction rule was defined as the combination of two or more clinical or biologic markers. Four investigators (CM, NG, SC, and OG) evaluated studies for possible inclusion. All studies were evaluated independently by at least two investigators. Nonrelevant studies were excluded based on title and abstract. For potentially relevant studies, the full text was obtained, and two investigators (CM, NG) independently assessed study eligibility and extracted the data on study design, patient characteristics, and outcomes. Disagreements were resolved by consensus or by discussion with a third reviewer (AP)

### Quality assessment

We used modified quality criteria based on the guidelines for assessing quality in prognostic studies [14]. Two investigators (CM, NG) assessed study quality independently. Each of six items was scored from 0 to 2. Studies with a total quality score between 11 and 12 were considered "good," between 9 to 10, "moderate," and 8 or less was considered "poor."

### Data analysis

For each score, the diagnostic performances (sensitivity, specificity, likelihood ratios, and diagnostic odds ratios) to predict different definitions of SCAP (ICU admission, early death, or intensive treatment) at the usual cut-off were pooled by using the method of the inverse of the variance. Random effects were systematically introduced [15]. Heterogeneity was measured by the I-square index [16] and tested with the Cochran test. Potential heterogeneity factors were explored by subgroup analyses for the Pneumonia Severity Index (PSI) (only score with more than 10 studies). A sensitivity analysis was conducted to check the robustness of the pooled sensitivities and specificities by removing each study, one by one. The R package "meta: Meta-analysis with R, version 1.6-1" was used for these analyses. For the PSI and CURB-65 (Confusion, Urea, Respiratory Rate, Blood pressure, Age > 65 years) scores, a summary ROC curve was assessed by the approach proposed by Moses *et al. *[17]. As several sensitivities and specificities were reported in the studies at different cut-offs, we used a linear mixed model with a correlation structure to take the dependence of the measures into account. The 95% confidence intervals of the areas under the curves were obtained by bootstrap. This analysis was performed with S-plus 8.0 for Windows. The significance level was 0.05 for all analyses. Forest plots of the Sensibility and Specificity were used for the graphic display of the results.

## Results

**Figure 1:**
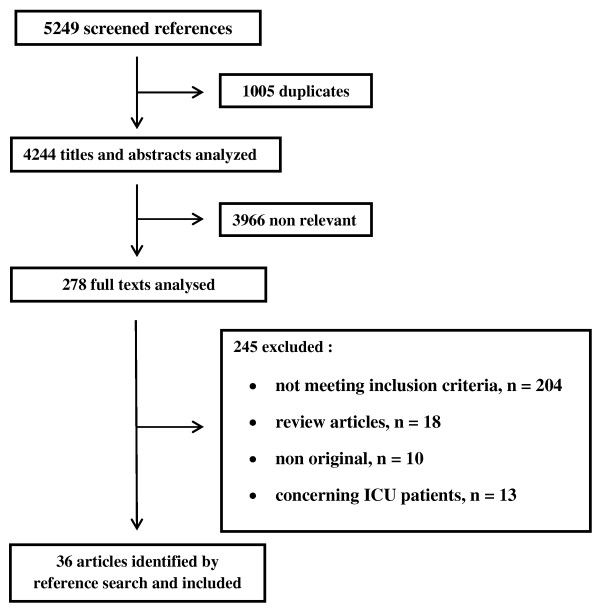
**Study flow chart**.

The search retrieved a total of 5,249 references, among which 1,005 duplicates were identified. Of the 4,244 remaining articles, 3,966 were excluded based on title and abstract (Figure 1). Full texts were obtained for the remaining 278 articles. Ten did not contain original data, 13 concerned only ICU patients, 204 did not meet inclusion criteria, 18 were review articles, and 33 satisfied inclusion criteria. Three articles [18-20] were identified by manual search of the references, leading to a total of 36 included articles [5,10-12,18-50]. Main characteristics of included studies are detailed in Table 1.

**Table 1:** Main characteristics of included studies

Study first author	Year of publication	Studied scores	End point	Prevalence end point	Uni- versus multicentric	Prospective/Retrospective	Ambulatory/Hospitalized	Number	NTBR	Major criteria excluded
Ananda-Rajah	2008	PSI/CURB-65	ICU admission	10.50%	Uni	R	H	408	Included	n

Angus	2002	PSI/ATS 2001/ATS 1993/URB	ICU admission	12.70%	Multi	P	H	1,339	Included	n

Brown	2009	IDSA-ATS 2007/SMART-COP/CURB-65/SCAP	IT in ICU/IT/ICU admission	12.35%	Uni	R	A+H	2,413	Excluded	n

Buising	2006	PSI/ATS 2001/CURB/CURB-65	ICU admission/death or ICU	6.60%	Uni	P	A+H	392	Included	n

Buising	2007	PSI/CURB/CURB-65/CORB	Vent/vaso ICU	6.30%	Uni	P	A+H	330	Included	n

Calbo	2004	PSI	ICU admission	3.30%	Uni	P	H	362	Included	n

Capelastegui	2006	CURB-65	ICU admission	4.09%	Uni	R	H	1,100	Included	n

Chalmers	2008	CRB-65/CURB-65	Vent/vaso	10.20%	Multi	P	A+H	1,007	Excluded	n

Chalmers	2011	IDSA-ATS 2007/SMART-COP/CURB-65/SCAP/CURB/CRB-65/PSI, ATS 2001 minor	Vent/vaso ICU admission	6.4% 7.6%	Uni	P	H	1,062	Excluded	y

Charles	2008	SMART-COP/CURB-65/PSI	Vent/vaso	10.30%	Multi	P	A+H	882	Included	n

Davis	2010	SMART-COP/SMARTACOP	Vent/vaso	21.00%	Uni	P	H	184	Excluded	n

Escobar	2008	Abbreviated PSI	ICU admission	12.30%	Uni	R	H	6,147	Included	n

Espana	2006	ATS 2001/PSI/CURB-65/SCAP	Death, vent, or septic shock	7.10%	Uni	P	A+H	1,057	Included	n

			Death, vent or septic shock	5.90%	Uni	P	A+H	719	Included	n

			Death, vent or septic shock	11.9%	Multi	R	H	1,121	Included	n

Espana	2010	SCAP/PSI/CURB-65	ICU admission	NA	Multi	R	H	712	Included	n

Ewig	2000	PSI	ICU admission	9%	Uni	P	H	232	Included	n

Ewig	2004	ATS 2001/PSI/CURB/CRB/URB/	ICU admission	16.70%	Uni	p	H	696	Excluded	n

Ewig	1998	ATS 1993/ATS 2001	ICU admission	16%	Uni	p	H	332	Excluded	n

Feagan	2000	PSI	ICU admission	13.60%	Multi	r	H	858	Included	n

Frei	2004	SBP/pH/O_2 _saturation/pulse	ICU admission	17%	Uni	r	H	782	Included	n

Fukuyama	2011	IDSA-ATS 2007/SMART-COP/CURB-65/SCAP/PSI/A-DROP	ICU admission Death, vent, or septic shock	7.6% 11.9%	Uni	p	H	505	Excluded	n

Garcia-Vidal	2008	PSI	Early death	2.3%	Uni	p	H	2,457	Included	

Garau	2008	PSI	ICU admission	5%	Multi	r	H	3,233	Included	n

Kamath	2003	CURB	ICU admission	10%	Uni	p	H	100	Excluded	n

Lamy	2004	PSI	ICU admission	14%	Uni	r	H	152	Included	n

Liapikou	2009	IDSA-ATS 2007/ATS 2001/PSI	ICU admission	11%	Uni	p	H	2,102	Excluded	n(y)

Man	2007	PSI/CURB-65/CRB-65/ATS 2001	ICU admission	4%	Uni	p	H	1,016	Included	n

Marrie	2007	PSI/CURB-65	ICU admission	10%	Multi	p	H	3,675	Included	n

Neill	1996	CURB	Mortality and ICU	11%	Uni	p	H	251	Excluded	n

Phua	2009	PSI/CURB-65/IDSA-ATS 2007	ICU admission	15%	Uni	p	H	1,017	Excluded	y

Putinati	2003	PSI	ICU admission	10%	Uni	p	H	229	Included	n

Renaud	2007	PSI	ICU admission	3.70%	Multi	p	H	566	Included	n

		PSI	ICU admission	10.50%	Multi	p	H	761	Included	n

Renaud	2009	REA-ICU	Early ICU admission < 3	4.40%	Multi	p	A+H	4,593	Excluded	y

Restrepo	2008	PSI/ATS 2001	ICU admission	19.90%	Multi	r	H	730	Excluded	n

Riley	2004	ATS 2001/PSI	ICU admission	23.69%	Uni	r	H	498	Excluded	n

Roson	2001	PSI	ICU admission	8%	Uni	p	H	533	Included	n

Shah	2010	CURB-65/PSI	ICU admission	23.30%	Uni	p	A+H	150	Included	n

Van der Eerden	2004	PSI	ICU admission	8.00%	Uni	p	H	260	Included	n

A, ambulatory; H, hospitalized; ICU, intensive care unit; IT, intensive treatment; P, prospective; R, retrospective; n, number; y, yes; NTBR, not to be resuscitated; Vent, ventilation; Vaso, vasopressor.

### Scores

**Figure 2:**
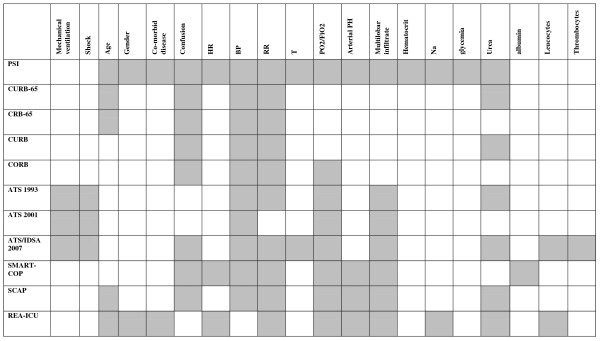
**Components of the main severity scores**. Criteria used in the score appear as shaded areas. BP, blood pressure; HR, heart rate; RR, respiratory rate; T, temperature.

We identified 11 main severity scores based on 20 variables. Components of the main severity scores are illustrated in Figure 2. Sufficient data were available to perform a meta-analysis on eight scores: PSI, CURB-65, CRB-65 (Confusion, Respiratory Rate, Blood pressure, Age > 65), CURB (Confusion, Urea, Respiratory rate, Blood pressure), ATS 2001 criteria, ATS/Infectious Disease Society of America (IDSA) 2007 criteria, SCAP score (Severe Community-Acquired Pneumonia), and SMART-COP (Systolic Blood pressure, Multilobar infiltrate, Albumin, Respiratory Rate, Tachycardia, Confusion, low Oxygen, low PH) (Table 2) Score definitions are included in the Additional file 1. Forrest plots for specificity and sensitivity of the eight scores for the prediction of ICU admission are provided in Additional file 2.

**Table 2:** Operative characteristics of the principal scores to predict ICU admission at their usual cut-off (95% CI)

	Sensitivity	Specificity	NLR	PLR	DOR
PSI ≥ 4	75.0 (71-78)	48.0 (44-52)	0.53 (0.46-0.6)	1.5 (1.4-1.6)	2.9 (2.4-3)

CURB-65 ≥ 3	56.2 (41-70)	74.2 (68-79)	0.64 (0.51-0.79)	2.1 (1.6-2.7)	3.6 (2.2-5.8)

CRB-65 ≥ 3	34.2 (18-55)	90.6 (89-92)	0.72 (0.54-0.97)	3.6 (1.9-6.9)	5.0 (2.0-12.7)

CURB ≥ 2	76.8 (48-92)	68.6 (53-81)	0.35 (0.18-0.70)	2.3 (1.9-2.7)	5.5 (3.7-8.2)

ATS 2001	69.5 (61-77)	90.1 (82-95)	0.37 (0.30-0.46)	7.3 (4.4-12.2)	24.6 (13.1-46.4)

ATS 2007	83.8 (48-97)	77.7 (46-93)	0.22 (0.08-0.66)	3.8 (1.7-8.6)	17.6 (13.1-24.1)

ATS 2007^a^	57.0 (46-68)	90.5 (84-95)	0.48 (0.38-0.6)	5.9 (3.8-9.3)	13.1 (7.7-22.3)

SCAP	93.8 (88-97)	45.6 (27-66)	0.13 (0.06-0.26)	1.8 (1.2-2.6)	14.9 (6.7-33.1)

SMART-COP	79.0 (69-87)	64.2 (30-66)	0.15 (0.03-0.91)	2.6 (1.3-5.3)	14.9 (8.6-25.7)

DOR, diagnostic odds ratio; NLR, negative likelihood ratio; PLR, positive likelihood ratio. ^a^Minor criteria.

#### PSI

**Figure 3:**
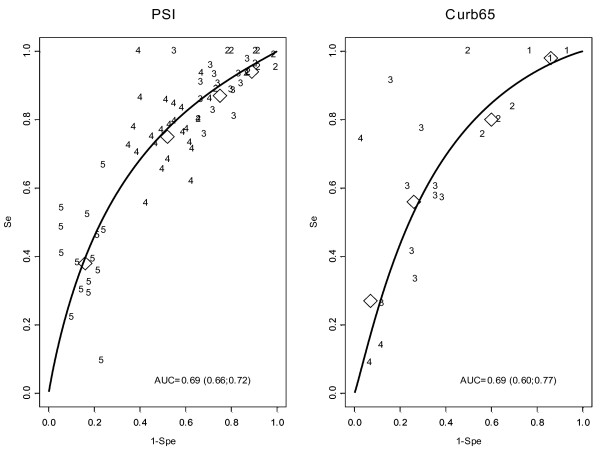
**SROC curve and area under the curve (AUC) of Pneumonia Severity Index (PSI) and CURB-65 to predict ICU admission**. Individual studies are represented by a number indicating the cut-off used. Their place on the diagram represents the sensitivity and specificity of the individual study. Diamonds represent meta-analytic test statistics for each cut-off.

Twenty-four studies [5,18,19,21,22,24-26,32,34,36,39-52], including 20,622 patients and 2,073 ICU admissions (10.1%), evaluated the performance of PSI to predict ICU need. A PSI score category of IV or more had a pooled sensitivity of 75% and a specificity of 48%. A cut-off of V increased specificity to 84% and decreased sensitivity to 38%. The global performance of PSI to predict ICU admission was modest, with an AUC of 0.69 (Figure 3). Significant heterogeneity was present. Performance of PSI to predict an alternative definition of SCAP, including mortality, was superior, with a pooled sensitivity of 92.4% (CI, 89 to 95) and specificity of 56.2% (CI, 43 to 69) in four cohorts including 3,195 patients [11,49].

#### CURB-65

CURB-65 was studied in nine cohorts [21,24,25,27,40,42,47,49,50] including a total of 5,773 patients and 479 ICU admissions (8.3%). At the usual cut-off value of 3 or more, pooled sensitivity was 56%, and specificity, 74%. The global performance of CURB-65 to predict ICU admission was similar to PSI with an AUC of 0.69 (Figure 3). Significant heterogeneity was present. The performance of CURB-65 to predict the need for ventilation or vasopressors was studied in three publications [10,28,50] including 2,951 patients, 264 requiring intensive treatment. Results were similar, with a pooled sensitivity of 57.2% (CI, 37 to 75) and specificity of 77.2% (CI, 73 to 81).

#### CRB-65

CRB-65 is a simplified version of the CURB-65 including only clinical predictors. Two studies [40,50] including 2,078 patients and 122 ICU patients (5.8%) calculated the performance of CRB-65 to predict ICU admission. For a threshold of 3 or more, pooled sensitivity was 34%, and specificity, 91%.

#### CURB (original BTS rule)

Performance of CURB to predict ICU admission was studied in four cohorts [24,25,32,38] totaling 1,418 patients and 161 ICU admissions (12.1%). Pooled sensitivity of a CURB score of 2 or more to predict ICU admission was 76.8%, and specificity, 68.6%. Significant heterogeneity was observed.

#### ATS 2001

The original ATS criteria for severe CAP published in 1993 [7] included 10 criteria. Some of these criteria were assessed at admission, and others, at any time during clinical course, limiting their use as a prediction rule. A new set of criteria was proposed by Ewig in 1998 [33] and adopted by the ATS in 2001. This prediction rule consists of two major (mechanical ventilation or shock) and three minor criteria (blood pressure < 90 mm Hg at admission, PaO_2_/FiO_2 _< 250 mm Hg, and multilobar involvement on chest radiograph). The prediction rule is considered positive in the presence of one major or two minor criteria.

**Figure 4:**
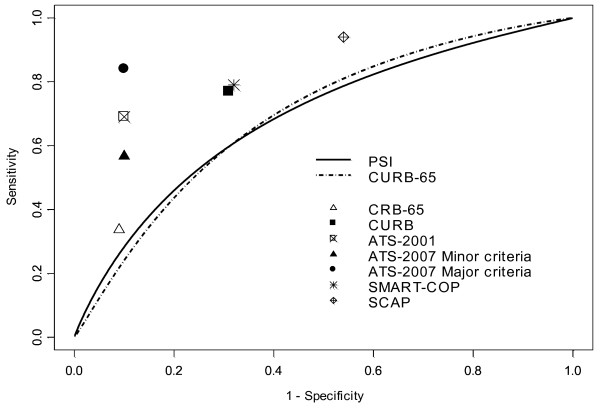
**Pooled discriminative performance of the principal scores for severe CAP compared with Pneumonia Severity Index (PSI) and CURB-65 ROC curve**.

We identified eight studies [5,22,24,32,33,39,40,45,49] including a total number of 7,116 patients with 908 ICU admissions (12.8%). The pooled sensitivity was 69.5%, and specificity, 90.1%. Pooled AUC could not be calculated because of insufficient data. Performance of the 2001 ATS criteria in comparison with PSI and CURB-65 is illustrated in Figure 4. A supplementary study [11] validated this rule to predict a composite definition of SCAP (in-hospital death, mechanical ventilation, or shock) in three cohorts including 2,897 patients and 252 SCAP (8.7%). Pooled sensitivity of the ATS 2001 criteria was 52.7%, and specificity, 95.1%. One study validated the use of the ATS 2001 minor criteria on a cohort excluding patients with therapeutic limitations or major criteria [50]. Sensitivity and specificity of two or more minor criteria to predict ICU admission were 47% and 91%.

#### ATS-IDSA 2007

A new prediction rule was proposed by the ATS and the Infectious Disease Society of America (IDSA) in 2007. This rule, known as the ATS-IDSA rule, consists of two major (mechanical ventilation or shock) and nine minor criteria (Figure 2). The rule is considered positive in the presence of one major or three minor criteria. We found five publications evaluating this rule [23,39,42,49,50]. Two studies, including 2,400 patients and 266 ICU patients (11%), validated the original rule to predict ICU admission [39,49]. Pooled sensitivity was 84%, and specificity, 78%.

Four studies [23,39,42,50] evaluated the performance of the minor criteria in a total of 6,412 patients including 650 ICU patients (10.1%). Pooled sensitivity was 57%, and specificity, 90%. Significant heterogeneity was present. Performance of the 2007 ATS-IDSA rule in comparison with PSI and CURB-65 is illustrated in Figure 4.

#### SMART-COP

Charles *et al. *[10] developed a prediction rule based on eight weighted criteria (see Additional File 1). This rule was validated in five external cohorts of pneumonia patients and further adapted and validated in two cohorts [29,50]. Pooled sensitivity of SMART-COP to predict the need for vasopressors or mechanical ventilation was 79%, and specificity, 68%.

Two studies evaluated this rule to predict ICU admission [49,50], with a pooled sensitivity of 79% and specificity of 64% on 1,567 patients including 112 ICU admissions (7.1%).

#### SCAP score

Espana *et al. *[11] derived and validated a prediction rule based on eight weighted criteria (see Additional File 1). Pooled performance of this rule on three cohorts totaling 3,402 patients (SCAP, 9%) to predict a composite definition of SCAP (in-hospital death, mechanical ventilation, or shock) was 92% (0.83 to 0.97) for sensitivity and 64% (0.5 to 0.76) for specificity.

Pooled performance of the SCAP score to predict ICU admission in two recent cohorts [49,50] was similar in terms of sensitivity (94%) but lower regarding specificity (46%)

### Other scores

Renaud *et al. *[12] proposed a prediction rule (REA-ICU index) based on 11 predictors (see Additional File 1). This rule was derived to predict early ICU admission (day 1 to day 3), excluding patients with an obvious ICU indication at admission and patients with therapeutic limitations (not to be resuscitated, NTBR order) and validated on four North American and European prospective multicenter cohorts including 6,560 patients. AUC was 0.81 (CI, 0.78 to 0.83) on the overall population.

An abbreviated version of the PSI was tested on an administrative database [30], and some authors proposed to modify the CURB rule to include an oxygenation parameter [25] (CORB). These scores have not been validated in other cohorts.

### Quality assessment and sources of heterogeneity

We completed quality assessment according to the criteria developed by Hayden *et al. *[14].

Nine studies were considered good (scores ≥ 11); 23, moderate (9 to 10); and four poor (≤ 8) quality (see Additional file 3). Important differences were observed in terms of design, populations, and ICU admission rates. Twenty-nine studies included only hospitalized patients, and seven included both hospitalized and ambulatory patients. Patients with therapeutic limitations (NTBR order) were excluded in 15 of 36 studies, and patients with an obvious indication for ICU, in three [12,42,47]. ICU admission rate varied from 3.3% [26] to 23.9% [45], and receipt of intensive treatment in the ICU was highly variable (16% to 100% [25,33]). When multiple measures of the same variable were available, as for vital signs, the measure introduced into the prediction rule was the first available one in four studies, the most abnormal one in five, and not specified in the majority of studies.

We performed an analysis to identify sources of heterogeneity for the only score (PSI) evaluated in at least 10 studies. Analyses did not reveal any significant factor of heterogeneity for sensitivity. Three factors influenced specificity: inclusion of NTBR patients decreased specificity; studies of good quality or high prevalence (≥ 15%) showed higher specificity. Nevertheless, the NTBR factor is highly correlated with the quality of the study (the NTBR patients were more frequently excluded in high-quality studies) and the prevalence (NTBR patients were more frequently excluded in studies with a high prevalence). NTBR exclusion is suspected to be the main factor explaining the heterogeneity observed in the specificity. The association between specificity and prevalence or quality may be caused by the correlation with NTBR exclusion.

Sensitivity analysis showed that the conclusions of the meta-analyses were robust and not caused by a single study. Sensitivity and specificity did not vary by more than 10%, except for CURB score: the study by Ewig [32] has a lower sensitivity and higher specificity than do other studies, but only four studies were included in this analysis.

## Discussion

In this systematic review of clinical prediction rules to predict severe CAP, PSI and CURB-65 have similar performances to identify patients requiring ICU admission. A PSI score of 4 or more is more sensitive (75% versus 56%) but less specific (47% versus 74%) than a CURB-65 score of 3 or more. These two scores, derived and validated to predict 30-day mortality, perform poorly to predict ICU admission, with an estimated AUC of 0.69. This performance is inferior to their original application: AUC for prediction of 30-day mortality was 0.80 in a recent systematic review [53].

Some explanations are available for this difference. These prediction rules, and especially the PSI, are heavily weighted by comorbidities and age and tend to consider as severe, patients in whom CAP is a terminal event. In many cohorts, the mortality rate is higher than the ICU admission rate, suggesting that an important proportion of patients with CAP die without being considered for ICU admission [21,39]. Age, a major component of these scores, is often negatively associated with ICU admission or intensive treatment [12,25,41] Exclusion of patients with therapeutic limitations may improve the specificity of these scores for ICU admission. In our heterogeneity analysis, PSI specificity increased from 45% to 56% when studies including NTBR patients were excluded (*P *= 0.008).

A new generation of scores, specifically developed to predict ICU admission, focuses on the severity of the pneumonia itself rather than on age and comorbid conditions. Overall, the performance of these scores seems superior to that of the PSI or CURB-65, particularly regarding specificity (90.1% and 90.5% for the ATS 2001 score and ATS/IDSA 2007 minor criteria versus 48.0% and 74.2% for PSI and CURB-65).

When considering scores derived over time, secular trends must be considered. Use of noninvasive ventilation (NIV) for severe CAP has increased over the last decade. This might have changed the proportion of patients receiving invasive mechanical ventilation. However, NIV was explicitly included in the definition of mechanical ventilation in most studies using that end point [10,25,29]. As the efficacy of NIV in alleviating respiratory failure for CAP patients is still debated, NIV is unlikely to have induced a major bias in the performance of the prediction rules over time. Furthermore, the principal scores (SCAP rule, SMART-COP, ATS 2007, and REA-ICU) have been proposed recently, at a time during which NIV for respiratory failure was routinely used.

Inclusion of major criteria in the ATS rules (mechanical ventilation and shock) improves their diagnostic performance but is not useful clinically, because these patients have an obvious indication for ICU admission. However, use of the minor criteria only does not seem to reduce the specificity of the ATS/IDSA 2007 score, as suggested by our results, with a pooled specificity of 90.5%. In a recent prospective cohort excluding patients with major criteria or therapeutic limitations, ATS-IDSA minor criteria had an AUC of 0.85 to predict ICU admission [50].

SMART-COP, REA-ICU, and SCAP scores seem to have operative characteristics similar to the ATS minor criteria but are less extensively validated. Also, confidence intervals are wide, and the use of different definitions of severe CAP limits comparison. Not surprisingly, these prediction rules share an important proportion of their predictive variables, as illustrated in Figure 2. Use of these three rules is more difficult than that of the ATS minor criteria, because of the weighting of the different predictive factors and the use of age-adjusted cut-offs.

Although the new generation of scores seems to have enhanced operative characteristics to predict ICU admission, their clinical utility is still debated. With a 10% prevalence of severe CAP and 57% sensitivity, even a specificity of 91% (as reached by the ATS-IDSA 2007 minor criteria) will lead to a positive predictive value of only 41%, leading to an important overuse of ICU resources. High negative predictive value is the most consistent finding among the different studies, suggesting that these scores could be more relevant to exclude the presence of a severe CAP than to aid in performing triage in patients for ICU admission. However, this high negative predictive value is mainly due to the low prevalence of severe patients. With a pooled sensitivity of 57%, the ATS-IDSA 2007 minor criteria would fail to identify almost one half of the patients with severe CAP, an unacceptably high proportion.

CAP is a complex and evolving inflammatory disease and critical clinical deterioration can result from various processes: respiratory failure, circulatory failure, destabilization of a preexisting comorbidity, appropriateness of initial antibiotic therapy, or hospital-acquired illnesses. It is not surprising that no single clinical rule has sufficient operating characteristics to be useful in this wide spectrum of evolution profiles.

The new generation of clinical prediction rules focuses on the early detection of respiratory and circulatory failure. Inclusion of various biomarkers such as procalcitonin [54], endothelin-1 [55], co-peptin [56], pro-atrial natriuretic peptide [57,58], or adrenomedullin [59] is hoped to improve this detection. Nevertheless, these biomarkers will probably fail to predict clinical deterioration due to hospital-acquired complications or decompensated comorbidities. It is even dubious whether they will detect circulatory or respiratory failure in patients admitted in the very early course of their disease.

In our view, rather than a definitive response to severity assessment, clinical prediction rules in patients with CAP should be considered an aid to clinical judgment particularly useful for less-experienced clinicians. Some of the included variables are consistently associated with a grim evolution, and their identification in an individual patient should alert for this possibility and trigger its timely reassessment and a thorough evaluation for intensive care or high-dependency-unit admission.

### Strengths

Our review incorporates the most recent published studies and updates the systematic review by Chalmers *et al. *[60]. We also used somewhat different inclusion criteria and data analysis. We did not include subgroups of patients based on age or pathogen, leading to the noninclusion of three studies [61-63]. Among the 40 studies included in the two systematic reviews, 24 were included in both, 12 in our work only, and four in the study by Chalmers *et al. *only. We decided against aggregating different definitions of SCAP, in an attempt to limit heterogeneity, and computed the performances of the prediction rules for the different definitions of SCAP. We included large recent studies [49,50] and could estimate the pooled performance of the more-recent prediction rules, SMART-COP and SCAP score. This allows direct comparison between recent prediction rules. Although included studies differed partially between our systematic review and that by Chalmers *et al.*, our final results are very similar, mutually strengthening their validity.

### Limitations

An important limitation of systematic reviews is their dependence on the quality of the included studies. Although the majority of included studies were considered of good or moderate quality, several pitfalls remain in the prediction of severe CAP. First, as already discussed, two biases are highly prevalent in these studies: inclusion in the studied population of patients not at risk for ICU admission (patients with therapeutic limitations); and use as a predictor of a surrogate of the outcome (use of mechanical ventilation and vasopressors, which are universally delivered only in an intensive or intermediate care unit).

Second, no universally accepted definition exists of severe CAP. The most frequently used proxy, ICU admission, is heavily influenced by ICU beds availability, local ICU admission policy, or subjectivity of the ICU specialist's evaluation. Use of a subjective decision such as ICU admission as a gold standard might lead to circular reasoning, because a perfect rule would be the one fitting usual practice. However, alternative definitions of SCAP, such as receipt of intensive treatment, do not seem to modify importantly operative characteristics of the prediction rules [23].

Third, definition of a "false positive" ICU admission is unclear: some patients might benefit from ICU admission even if they are not receiving vasopressors or mechanical ventilation (for example, through better fluid resuscitation).

Fourth, some of the studied rules have been fully incorporated in expert society recommendations. This might lead to contamination of ICU admission practices, further leading to an overestimation of their accuracy.

Finally, major heterogeneity was present among included studies, limiting the validity of the meta-analysis.

## Conclusions

PSI and CURB-65 do not have sufficient operating characteristics to be useful for making ICU triage decisions in severe CAP. Newer rules, specifically conceived to aid in identifying severe CAP, perform better but still have insufficient test characteristics to be a major help in everyday decisions. Recent clinical prediction rules should be considered an aid to clinical judgment to guide ICU admission in CAP patients. Clinical trials evaluating this issue should exclude patients who are not candidates for ICU admission and predicting factors that make ICU admission mandatory. Inclusion of new biomarkers, dynamic reassessment of the severity scores, and impact studies evaluating their use would deserve evaluation in future clinical research.

## Key messages

• Identification of severe community-acquired pneumonia (SCAP) should allow admission of patients at the appropriate level of care.

• Traditional severity scores, the PSI and CURB 65, perform poorly to identify patients requiring ICU admission.

• New dedicated scores have better operative characteristics and could be useful adjuncts to clinical judgment.

## Abbreviations

ATS: American Thoracic Society; AUC: area under the curve; CAP: community-acquired pneumonia; CURB: confusion, urea, respiratory rate, blood pressure; ICU: intensive care unit; IDSA: Infectious Disease Society of America; NIV: noninvasive ventilation; NTBR: not-to-be resuscitated; PSI: pneumonia severity index; ROC: receiver operating characteristic; SCAP: severe community-acquired pneumonia; SMART-COP: systolic blood pressure, multilobar infiltrate, albumin, respiratory rate, tachycardia, confusion, low oxygen, low pH.

## Competing interests

The authors declare that they have no competing interests.

## Authors' contributions

APe, CM, and NG designed the study. CM, NG, OG, and SC collected the data. APo and CC performed the statistical analysis. CM and NG wrote the draft. All authors critically revised the manuscript and approved the final version for publication.

## Supplementary Material

Additional file 1 **Definition of the different scores**. This file contains the detailed components and cut-offs values of the different prediction rules (PSI, CURB-65, ATS-2001, IDSA/ATS 2007, SCAP score, SMART-COP, and REA-ICU). Click here for file

Additional file 2 **Forrest plots of sensitivity/specificity of the different scores to predict ICU admission**. This file contains the Forrest plots of the eight meta-analyzed scores (PSI, CURB-65, CURB, CRB-65, SMART-COP, SCAP score, ATS-2001, and IDSA/ATS 2007) for the outcome ICU admission. Click here for file

Additional file 3 **Study quality assessment**. Global and detailed quality assessment for each included study is provided in this file. Click here for file
