# Supplementary material for: Prediction of severe community-acquired pneumonia: a systematic review and meta-analysis
Source: Crit Care. 2012 Jul 27;16(4):R141. doi: 10.1186/cc11447 (PMC3580727; doi:10.1186/cc11447)
Supplement: Additional file 1 — Definition of the different scores. This file contains the detailed components and cut-offs values of the different prediction rules (PSI, CURB-65, ATS-2001, IDSA/ATS 2007, SCAP score, SMART-COP, and REA-ICU). [file cc11447-S1.PDF]

**Additional file 1:**

**Definition of the different scores**

**1. Pneumonia Severity Index (PSI)**

| **Risk factor** | **Points assigned** |
|---|---|
| **Demographics** | |
| Men | Age (years) |
| Women | Age (years) -10 |
| **Comorbidities** | |
| Nursing home resident | +10 |
| Neoplasm | +30 |
| Liver disease | +20 |
| Heart failure | +10 |
| Stroke | +10 |
| Renal failure | +10 |
| **Physical examination findings** | |
| Altered mental status | +20 |
| Respiratory rate > 30 per minute | +20 |
| Systolic blood pressure < 90 mmHg | +20 |
| Temperature < 35 °C or ≥ 40 °C | +15 |
| Pulse rate >125 per minute | +10 |
| **Laboratory and radiographic findings** | |
| Arterial PH < 7.35 | +30 |
| Blood urea nitrogen >30mg/dl | +20 |
| Sodium < 130 mmol per L | +20 |
| Glucose ≥ 250 mg per dL | +10 |
| Hematocrit < 30 percent | +10 |
| PaO2 < 60 mmHg | +10 |
| Pleural effusion | +10 |
| **Total points** | |

Patients with PSI score ≥ 91 (Class ≥ IV) should be considered for ICU admission

**2. CURB-65 /CRB65/CURB**

| **Predictor** | **Point assigned** |
|---|---|
| **C**onfusion | 1 |
| **U**rea > 7mmol/l | 1 |
| **R**espiratory rate ≥ 30/min | 1 |
| Systolic **B**lood pressure < 90 mmHg or<br>Diastolic **B**lood pressure < 60 mmHg | 1 |
| Age > **65** years | 1 |

Patients with a CURB65 score of ≥3 should be considered for ICU admission. CRB65 and CURB are simplified versions of the CURB65. Patients with CURB and CRB-65 ≥2 should be considered for ICU admission.

**3. ATS 2001 Criteria**

MAJOR CRITERIA

- Requirement for mechanical ventilation
- Septic shock

MINOR CRITERIA

- Systolic blood pressure <90mmHg
- Multilobar chest x-ray infiltrates
- PaO2/Fi02 <250 mmHg

ICU admission is recommended for patients who meet 1 of 2 major criteria or 2 of 3 minor criteria.

**4. ATS-IDSA 2007 Criteria**

MAJOR CRITERIA

- Invasive mechanical ventilation
- Septic shock with the need for vasopressors

MINOR CRITERIA

- Respiratory rate $\geq$ 30 breaths per minute
- PaO2/FiO2 ratio <250mmHg
- Multilobar chest X ray infiltrates
- Confusion/disorientation
- Uremia >20mg/dl (7 mmol/ L)
- Leukocytes <4000 /$mm^3$
- Thrombocytes <100,000 /$mm^3$
- Hypothermia (temperature <36 degrees C)
- Hypotension requiring aggressive fluid resuscitation

ICU admission is recommended for patients who meet 1 of 2 major criteria or 3 of 9 minor criteria

**5. SMART-COP**

| **Predictor** | **Point assigned** |
|---|---|
| **S**ystolic BP < 90 mmHg | 2 |
| **M**ultilobar chest x-ray involvement | 1 |
| **A**lbumin <35g/l | 1 |
| **R**espiratory rate<br>- Age $\leq$ 50 years = $\geq$ 25/mn<br>- Age >50 years = $\geq$ 30/mn | 1 |
| **T**achycardia > 125/min | 1 |
| **C**onfusion | 1 |
| **O**xygenation<br>- Age $\leq$50 years= <70mmHg or $O_2$ sat $\leq$93%<br>- Age >50 years= <60mmHg or $O_2$ sat $\leq$90% (2 point) | 2 |
| **P**H <7.35 (arterial) | 2 |

ICU admission is recommended for patients with scores > 5 points.

**6. SCAP Prediction rule (or CURXO 80)**

| **Predictor** | **Points assigned** |
|---|---|
| Arterial pH < 7.30 | 13 |
| Systolic blood pressure< 90mmHg | 11 |
| Respiratory rate > 30 /min | 9 |
| Altered mental status | 5 |
| Blood urea nitrogen > 30 mg/dL | 5 |
| Oxygen arterial pressure < 54 mmHg PaO2 /FiO2 < 250 mmHg | 6 |
| Age ≥ 80 years | 5 |
| Multilobar / bilateral lung affectation | 5 |

ICU admission is considered for a score of 10 or more

**7. REA-ICU**

| **Predictor** | **Point assigned** |
|---|---|
| Male gender | 1 |
| Co-morbid condition | 1 |
| Respiratory rate > 30/min | 1 |
| Leucocytes <3 or > 20 G/L | 1 |
| Heart Rate > 125/min | 1 |
| Age < 80 | 1 |
| Multilobar infiltrate or pleural effusion | 2 |
| Oxygen saturation <90% or $Pa0_2$ <60mmHg | 2 |
| Arterial pH < 7.35 | 2 |
| Blood urea nitrogen $\geq$ 11mmol/L | 2 |
| Sodium < 130 mmol/L | 3 |

This rule was derived and validated to predict early (< 3 days) ICU admission.

No clear cut-off value was proposed. Patients with 7 or 8 points have a 11-12.25% risk of early ICU admission and patients with 9 points or more 27.1-32.4%.
