# Supplementary material for: Prediction of severe community-acquired pneumonia: a systematic review and meta-analysis
Source: Crit Care. 2012 Jul 27;16(4):R141. doi: 10.1186/cc11447 (PMC3580727; doi:10.1186/cc11447)

Additional file 2

Forest Plots of sensitivity and specificity meta-analysis for the outcome ICU admission

Figure 1: Sensitivity of PSI ≥ 4 to predict ICU admission

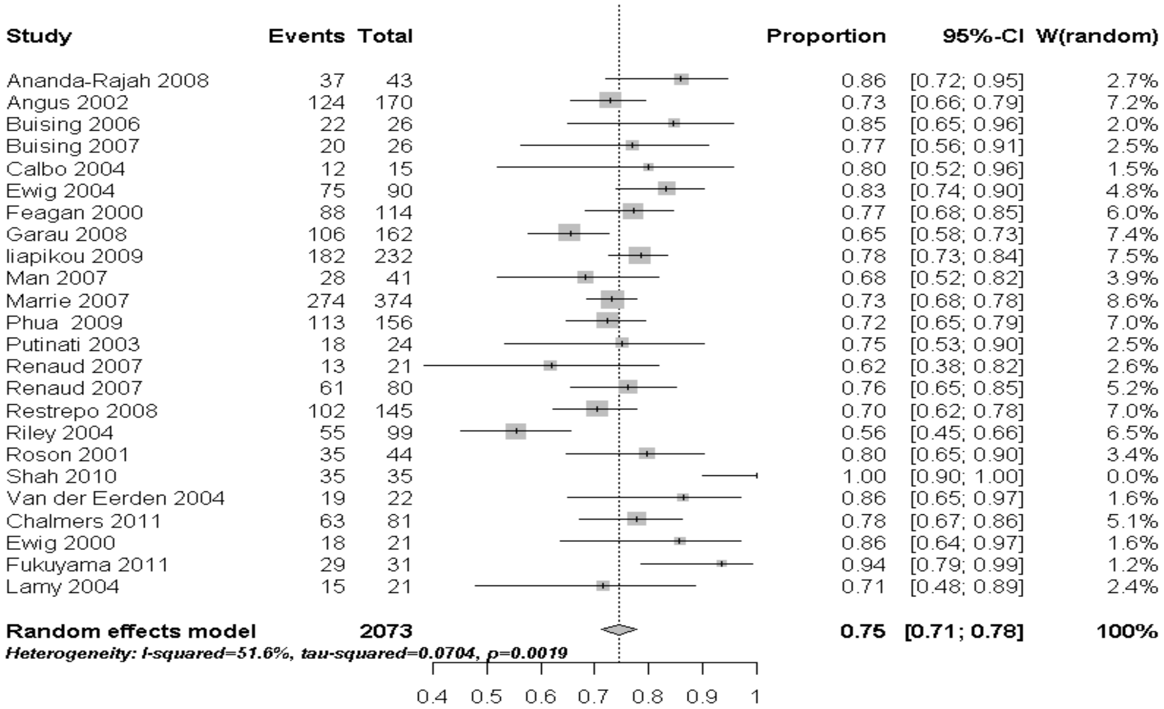

Figure 2: Specificity of PSI ≥ 4 to predict ICU admission

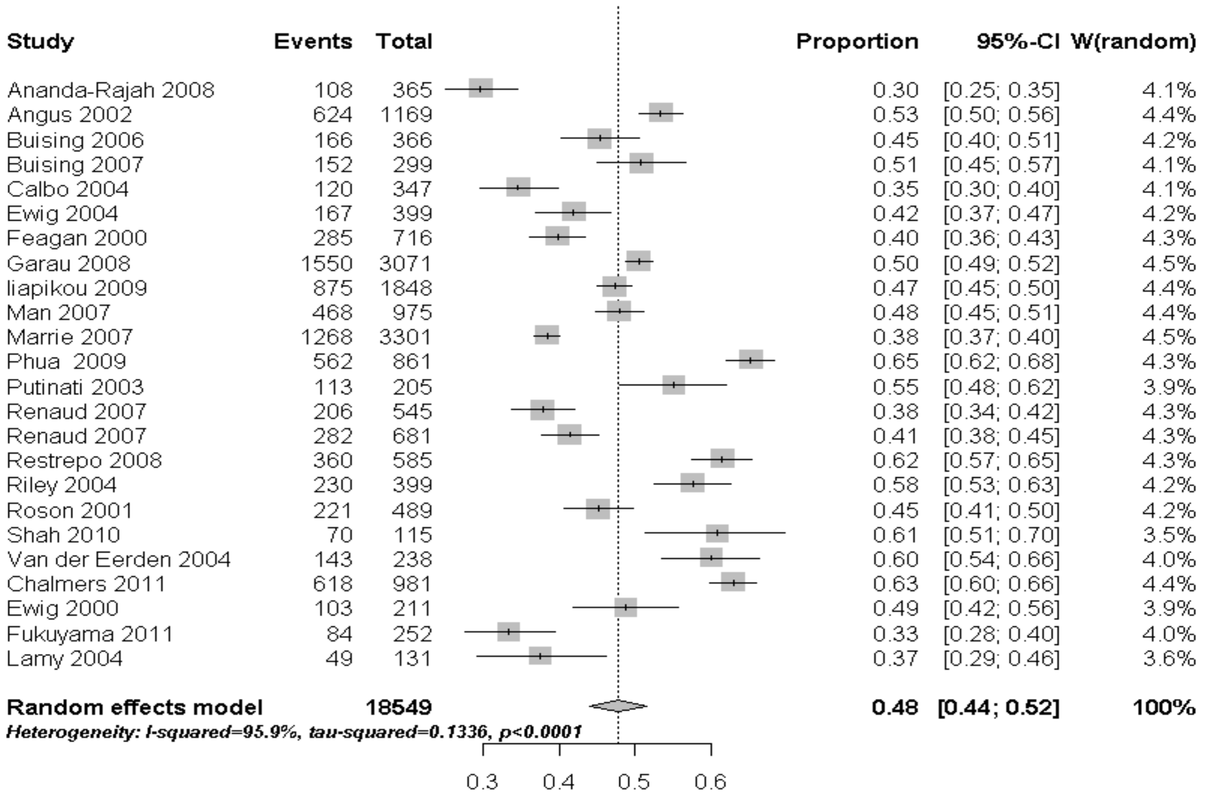

Figure 3: Sensitivity of CURB-65 score ≥ 3 to predict ICU admission

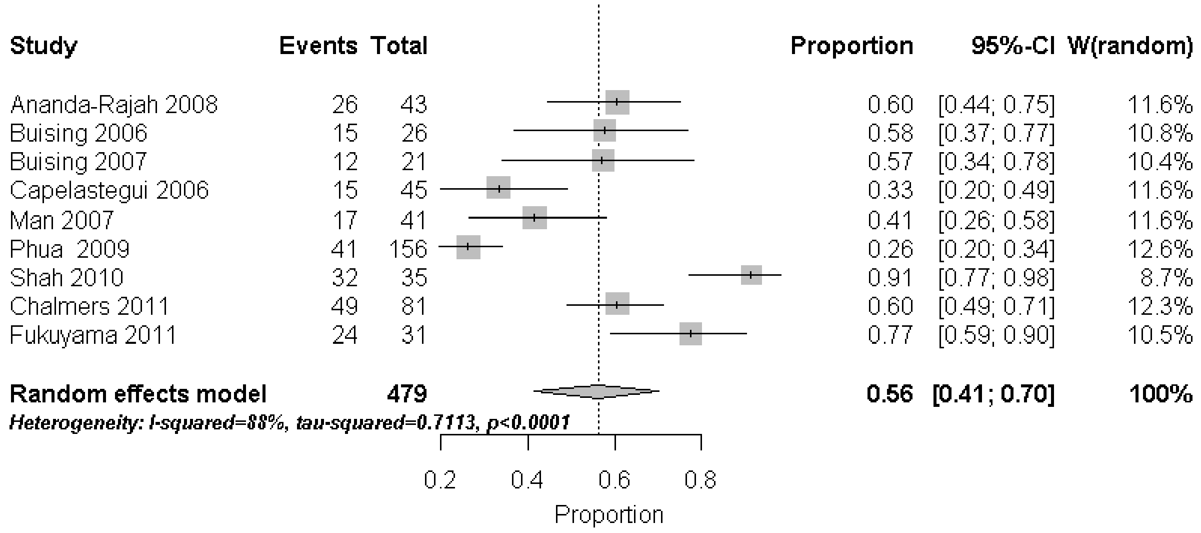

Figure 4: Specificity of CURB-65 score ≥ 3 to predict ICU admission

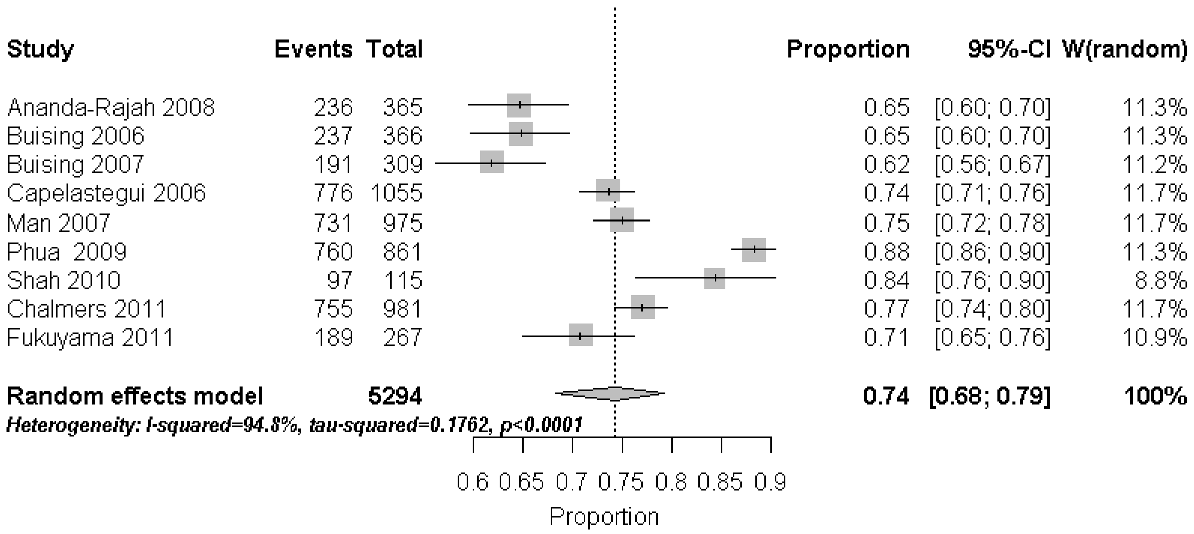

Figure 5: Sensitivity of CRB-65 score ≥ 3 to predict ICU admission

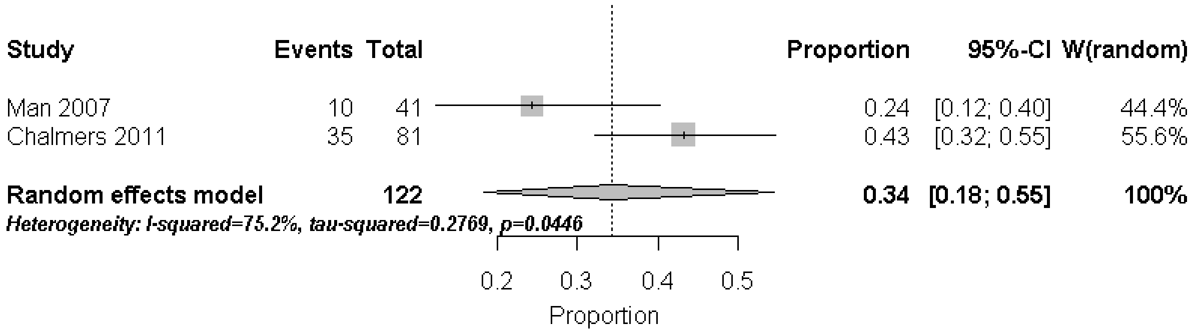

Figure 6: Specificity of CRB-65 score ≥ 3 to predict ICU admission

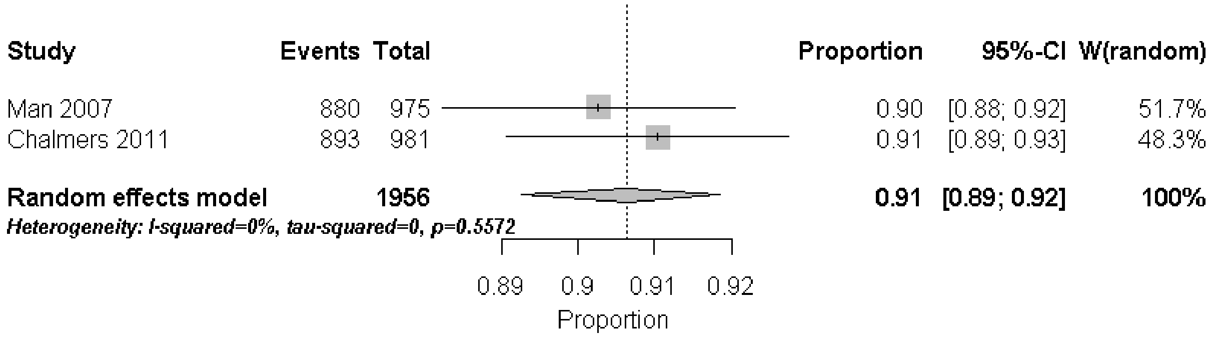

Figure 7: Sensitivity of CURB score ≥ 2 to predict ICU admission

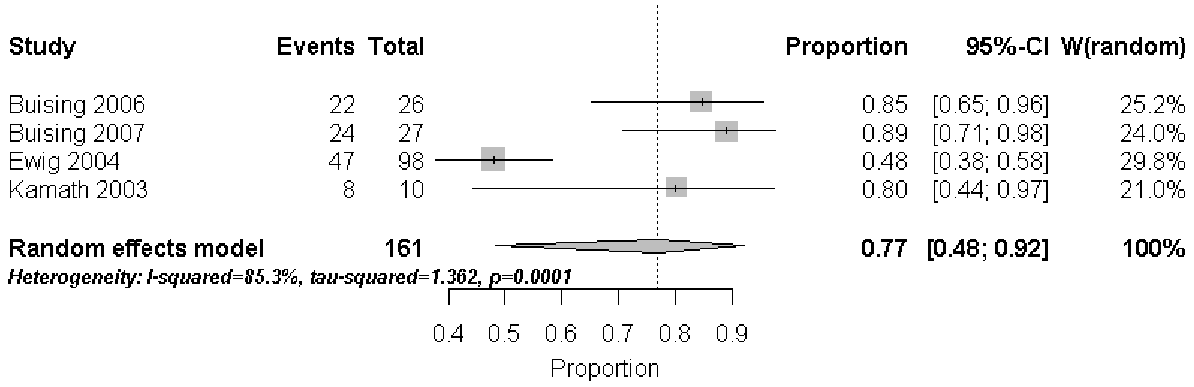

Figure 8: Specificity of CURB score ≥ 2 to predict ICU admission

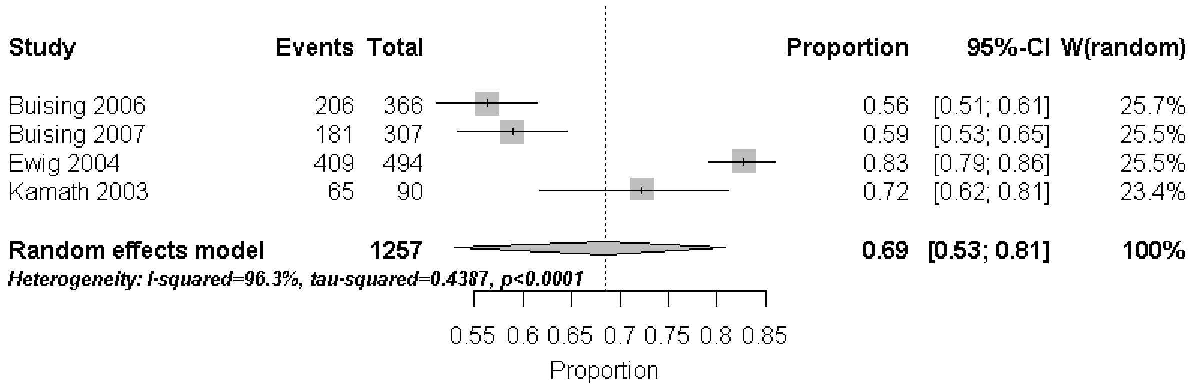

Figure 9: Sensitivity of ATS 2001 score to predict ICU admission

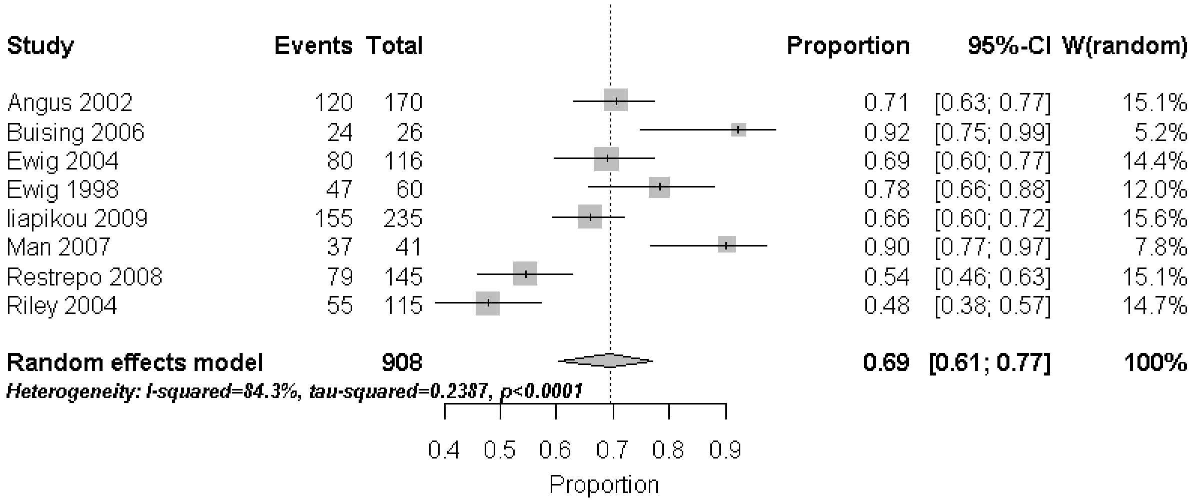

Figure 10: Specificity of ATS 2001 score to predict ICU admission

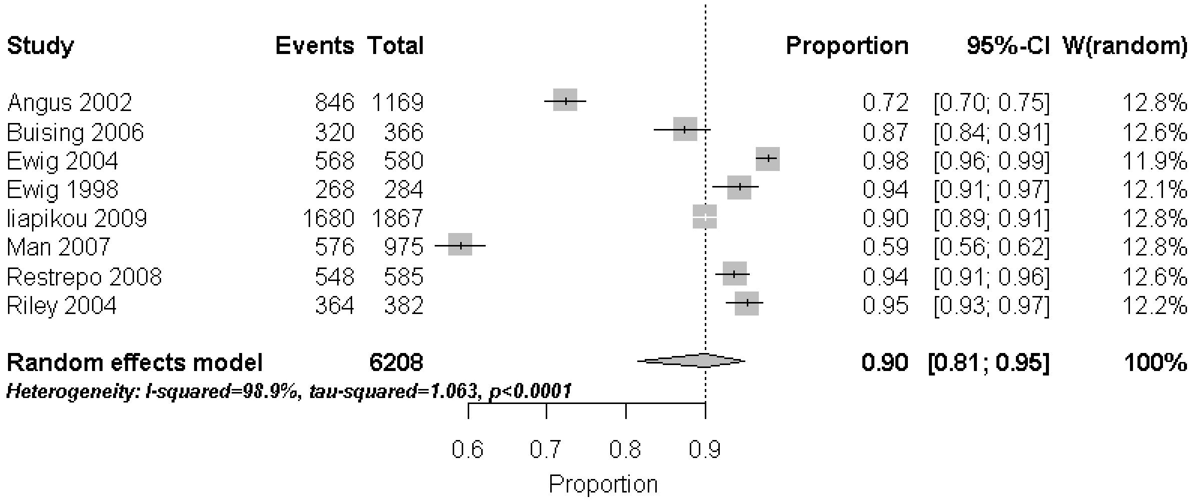

Figure 11: Sensitivity of IDSA-ATS 2007 score to predict ICU admission

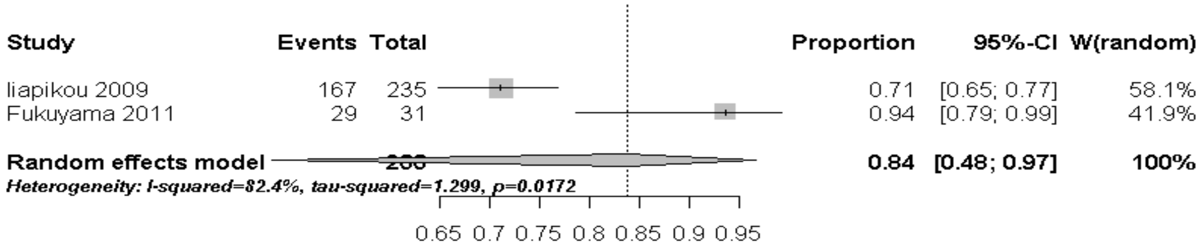

Figure 12: Specificity of IDSA-ATS 2007 score to predict ICU admission

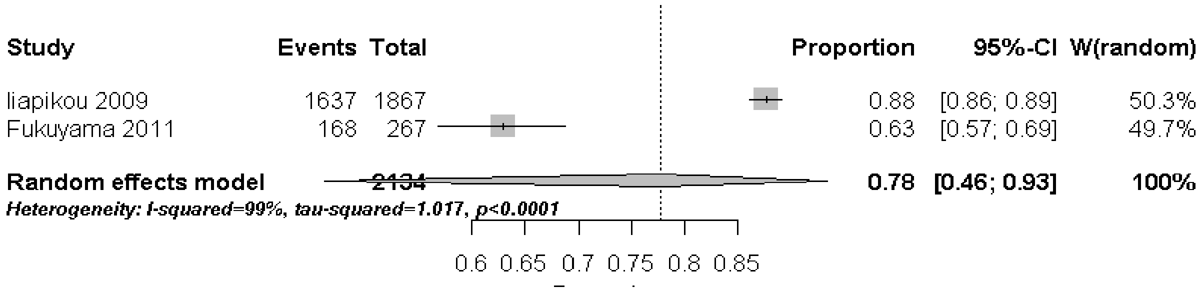

Figure 13: Sensitivity of IDSA-ATS 2007 ≥3 minor criteria to predict ICU admission

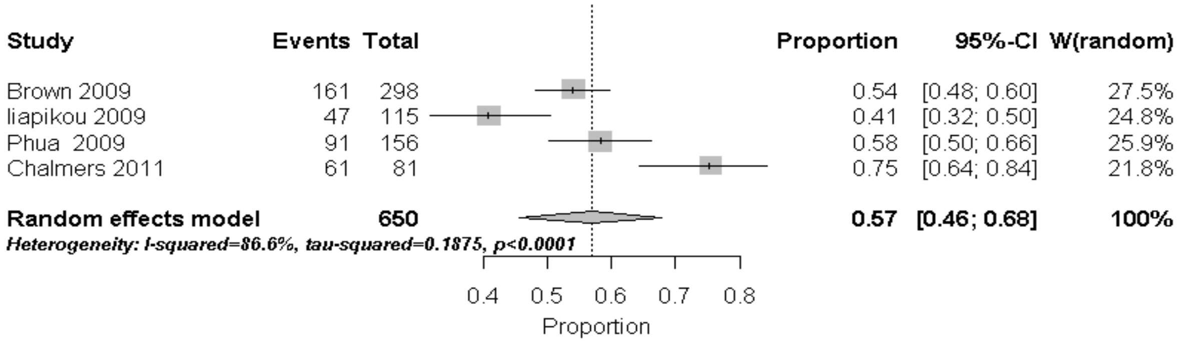

Figure 14: Specificity of IDSA-ATS 2007 ≥3 minor criteria to predict ICU admission

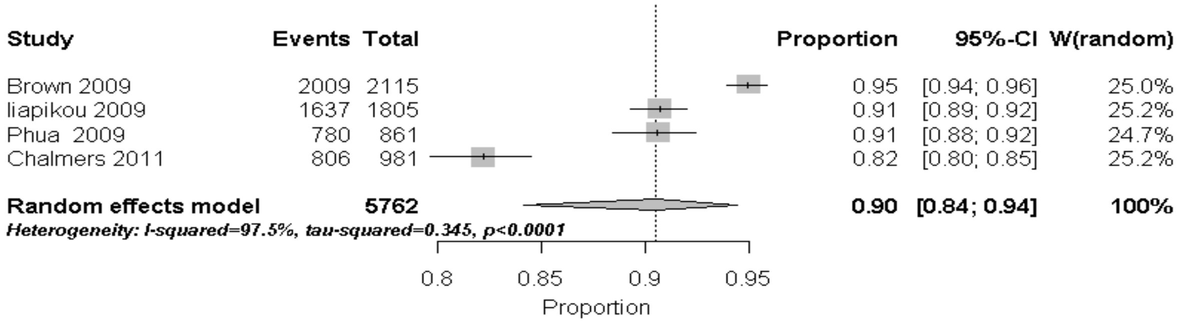

Figure 15: Sensitivity of SMART-COP to predict ICU admission

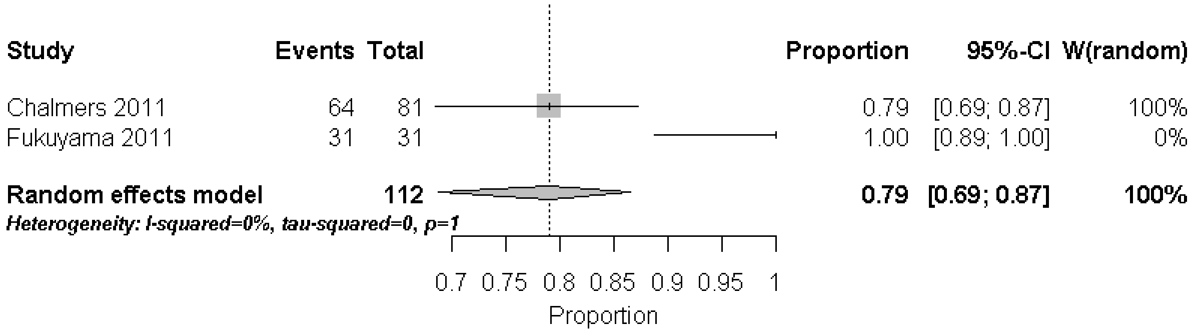

Figure 16: Specificity of SMART-COP to predict ICU admission

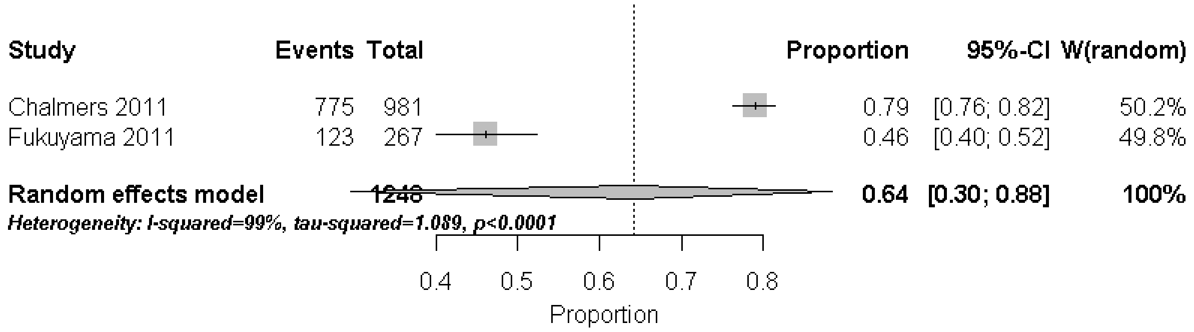

Figure 17: Sensitivity of SCAP to predict ICU admission

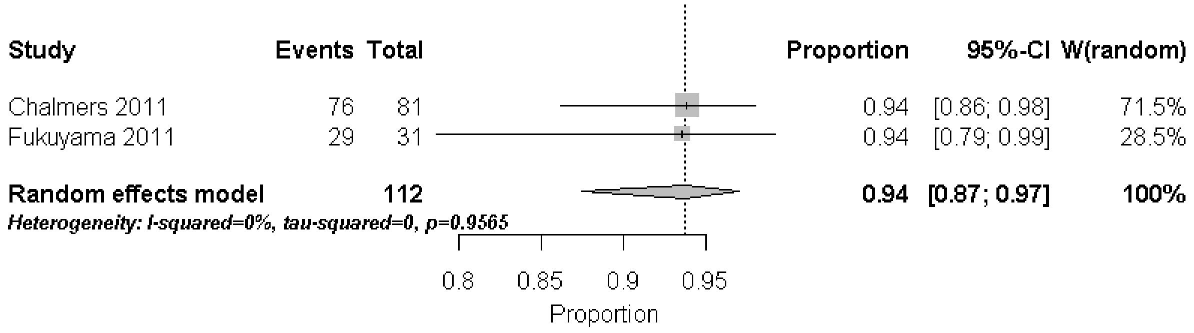

Figure 18: Specificity of SCAP to predict ICU admission

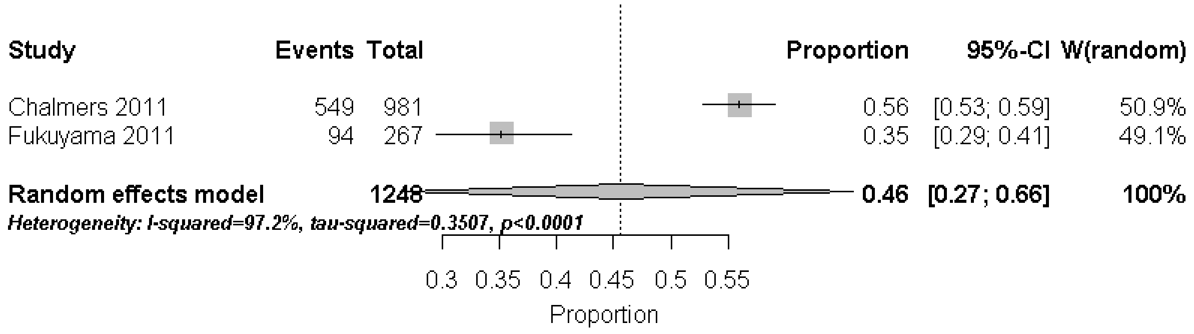

Supplement: Additional file 2 — Forrest plots of sensitivity/specificity of the different scores to predict ICU admission. This file contains the Forrest plots of the eight meta-analyzed scores (PSI, CURB-65, CURB, CRB-65, SMART-COP, SCAP score, ATS-2001, and IDSA/ATS 2007) for the outcome ICU admission. [file cc11447-S2.PDF]
