# Supplementary material for: Prediction of severe community-acquired pneumonia: a systematic review and meta-analysis
Source: Crit Care. 2012 Jul 27;16(4):R141. doi: 10.1186/cc11447 (PMC3580727; doi:10.1186/cc11447)
Supplement: Additional file 3 — Study quality assessment. Global and detailed quality assessment for each included study is provided in this file. [file cc11447-S3.PDF]

Additional file 3:

Study quality assessment

Study	Participation	Attrition	Prognostic factor measurement	Outcome measurement	Confounding measurement	Analysis	Quality score (total)
Ananda-Rajah 2008	2	1	1	2	1	2	9
Angus 2002	2	2	2	2	1	2	11
Brown 2009	2	2	1	2	1	1	9
Buising 2006	2	2	2	2	2	2	12
Buising 2007	1	2	2	2	1	2	10
Calbo 2004	1	2	1	1	1	2	8
Capelastegui 2006	2	2	1	2	1	2	10
Chalmers 2008	2	2	1	1	2	2	10
Chalmers 2011	2	2	1	2	2	2	11
Charles 2008	2	2	2	1	2	2	11
Davis 2010	1	2	2	1	2	2	10
Escobar 2008	1	2	1	1	1	2	8
Espana 2006	2	2	1	1	1	2	9
Espana 2010	2	2	1	1	1	1	8
Ewig 2000	2	2	1	2	1	2	11
Ewig 2004	2	2	2	2	1	2	11
Ewig 1998	2	2	1	2	1	2	10
Feagan 2000	2	2	1	2	1	2	10
Frei 2004	2	2	1	2	1	2	10
Fukuyama 2011	2	2	1	2	1	2	10
Garau 2008	2	2	1	2	1	2	10
Garcia-Vidal 2008	2	2	1	1	1	2	10
Kamath 2003	1	2	1	2	1	2	9
Lamy 2004	1	2	1	2	1	1	8
liapikou 2009	2	2	1	2	1	2	10

Man 2007	2	2	1	2	1	2	10
Marrie 2007	2	2	1	2	1	2	10
Neill 1996	2	2	1	1	1	2	9
Phua 2009	2	2	2	2	1	2	11
Putinati 2003	2	2	1	1	1	2	9
Renaud 2007	2	2	1	2	1	2	10
Renaud 2009	2	2	1	2	1	2	10
Restrepo 2008	2	2	1	2	1	1	9
Riley 2004	2	2	2	2	1	2	11
Shah 2010	1	2	1	2	1	2	9
Shin 2007	2	2	2	1	1	2	10
Van der Eerden 2004	2	2	2	2	1	2	11
